# Effects of dexmedetomidine on cognitive function in elderly patients after laparoscopic cholecystectomy: A protocol for systematic review and meta-analysis

**DOI:** 10.1097/MD.0000000000020177

**Published:** 2020-05-15

**Authors:** Na Li, Lu Xiong, Ye-Hua Wu, Xiao-Jian Chen, Ya-Zhen Meng, Shuang-Feng Li, Ya-Qin Xiong

**Affiliations:** a Department of Anesthesiology, Hainan General Hospital (Hainan Affiliated Hospital of Hainan Medical University), Haikou, Hainan; b Department of Anesthesiology, West China Second University Hospital of Sichuan University, Chengdu, Sichuan, China.

**Keywords:** cognitive function, dexmedetomidine, effects, elderly patients, laparoscopic cholecystectomy, safety

## Abstract

**Background:**

Although many studies have reported the effects of dexmedetomidine on cognitive function (CF) in elderly patients after laparoscopic cholecystectomy (LCT), to this date, its effects are still not well understood. The aim of this study is to produce a qualitative synthesis of assessing the effects of dexmedetomidine on CF in elderly patients after LCT.

**Methods:**

We will conduct a comprehensive search in Cochrane Library, MEDLINE, EMBASE, CINAHL, PsycINFO, Scopus, VIP Database, WANGFANG Database, Chinese Biomedical Literature Database, and China National Knowledge Infrastructure from the commencement to March 31, 2020 without restrictions of language and publication status. In addition, we will also search grey literature, including conference abstracts, dissertations, reference lists of included studies and relevant reviews. All potential studies will be identified independently by 2 authors to determine their inclusion against previously defined eligibility criteria. The quality of selected papers will be assessed using Cochrane risk of bias tool. All statistical analysis will be performed using RevMan 5.3 software.

**Results:**

This study will provide a synthesis of the current available data on assessing the effects of dexmedetomidine on CF in elderly patients after LCT.

**Conclusions:**

Its findings will provide qualitative evidence to better understand the effects of dexmedetomidine on CF in elderly patients after LCT.

**INPLASY Registration Number:** INPLASY202040030.

## 1 Introduction

Laparoscopic cholecystectomy (LCT) is undertaken with increasing frequency among elderly populations.^[1,2,3,4,5,6]^ In addition, many surgeons consider LCT as a relatively simple management procedure which can help patient recovery quickly, and it accounts for 25% to 30% of all surgical procedures in elderly treatments.^[7,8]^ However, postoperative cognitive function (CF) impairment often accompanies LCT in the elderly population.^[1,9,10,11]^ Therefore, it is very important to manage CF impairment after LCT.

Dexmedetomidine is a highly selective α2-receptor agonist that is associated with excellent sedative and analgesic effects with minor respiratory depression.^[12,13,14,15,16]^ Recent studies indicate that it has excellent sedation and analgesia and results in greater patient satisfaction in LCT procedures in the elderly patients.^[17,18,19,20,21,22,23,24,25]^ Additionally, there are contrast results among those trials. Thus, this study will perform a systematic review to assess the efficacy and safety of dexmedetomidine on CF in elderly patients after LCT.

## 2 Methods

### 2.1 Study registry

This study is registered through INPLASY202040030. It has been reported following the Preferred Reporting Items for Systematic review and Meta-Analysis Protocols.^[26]^

### 2.2 Eligibility criteria for including studies

#### 2.2.1 Types of participants

This study will include elderly participants (over 65 years old) who underwent LCT and had CF regardless their countries, races and sex.

#### 2.2.2 Types of studies

This study will only include randomized controlled trials (RCTs) that focus on investigating the effects of dexmedetomidine on CF in elderly patients after LCT. We will exclude any other studies, such as non-clinical trials, case report, case series, and non-RCTs.

#### 2.2.3 Types of interventions

In the experimental group, all participants received dexmedetomidine intervention for their managements.

In the control group, patients underwent any treatments, including anesthetic medication or alternative therapies, except dexmedetomidine.

#### 2.2.4 Types of outcome measurements

The primary outcome includes cognitive disorder changes. It can be assessed by any relevant scales, such as Modified Mental State Examination scale.

The secondary outcomes consist of pain intensity (checked by any pain scales); short-term memory (measured by any associated tools, such as short-term memory summary score); quality of life (assessed by any relevant scales, such as activities of daily living); and adverse events.

### 2.3 Data sources and search

The search will be performed in Cochrane Library, MEDLINE, EMBASE, CINAHL, PsycINFO, Scopus databases, VIP database, WANGFANG database, Chinese Biomedical Literature Database, and China National Knowledge Infrastructure. All these electronic databases will be searched from the commencement to the March 31, 2020 with no limitations of language and publication status. We will create a search strategy sample for MEDLINE in Table 1. We will also present similar search strategies for other electronic databases.

**Table 1:**
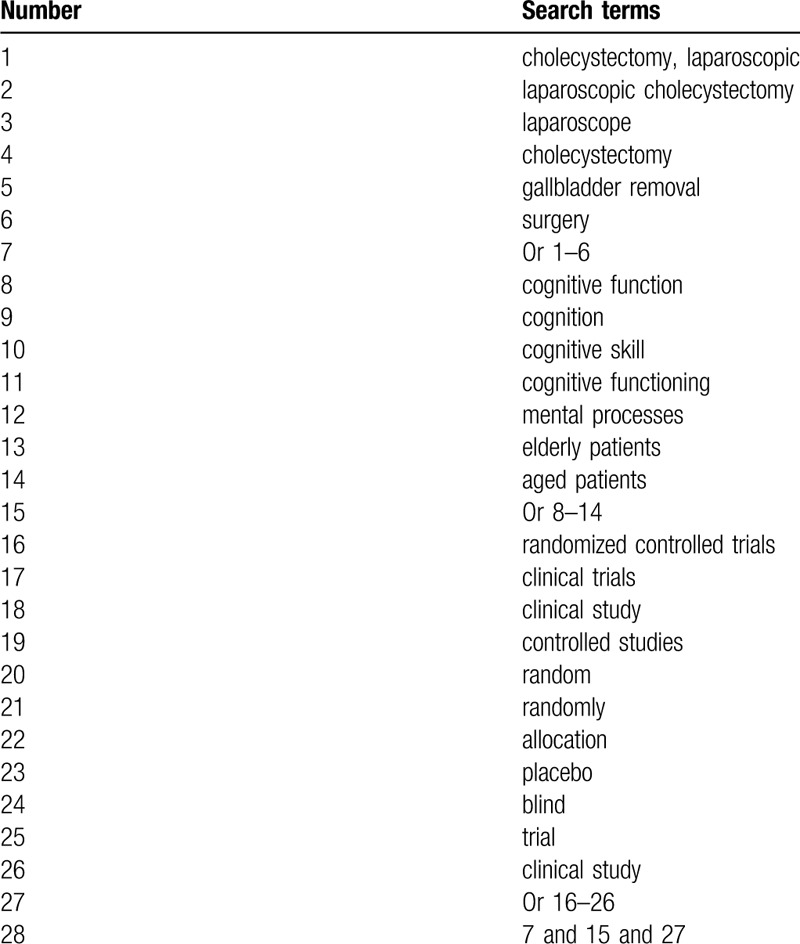
Detailed search strategy of MEDLINE.

Besides the above sources, we will also check grey literature, such as conference abstracts, dissertations, reference lists of included studies and relevant reviews.

### 2.4 Study selection

The titles and abstracts of all identified citations will be scanned by 2 independent authors according to the predefined eligibility criteria. All irreverent studies will be removed. Then, we will obtain full-papers of remaining studies against the all inclusion criteria. If we detected any divergences between 2 authors, we will invite a third author to solve them through discussion. We will present the whole process of study selection in a flow chart.

### 2.5 Data extraction

We will create a standardized data collection sheet for data extraction before we perform it. Two authors will independently extract general descriptive data: authors, year of publication, country, language, title, characteristics of patients, diagnostic criteria, inclusion and exclusion criteria, number of patients, study design, study methodology, details of interventions and controls (such as deliver mode, frequency, dosage, duration), outcomes, safety, and any other relevant information. Any divergences occur between 2 authors regarding the data extraction, and a third author will be invited to resolve these issues by discussion.

### 2.6 Missing data dealing with

If we find any unclear or missing information, we will contact primary authors to obtain them. If we cannot obtain those data, we will only analyze data at hand, and will report its impact as a limitation.

### 2.7 Risk of bias assessment

Risk of bias for each included study will be evaluated by two independent authors using Cochrane risk of bias tool. It will assess each trial through 7 aspects and each one will be graded as high risk of bias, unclear risk of bias, or low risk of bias. Any opposition between 2 authors will be solved by a third author through consultation.

### 2.8 Subgroup analysis

When necessary, we will undertake a subgroup analysis to investigate the potential heterogeneity and inconsistency based on the different patient characteristics, study quality, treatments, controls, and outcome measurements.

### 2.9 Sensitivity analysis

If necessary, we will also perform a sensitivity analysis to identify the stability of merged outcome data by excluding studies with high risk of bias.

### 2.10 Reporting bias

If more than 10 eligible trials are entered, we will carry out funnel plot and Egger regression test to check if there is reporting bias in this study.^[27,28]^

### 2.11 Data synthesis

RevMan 5.3 software will be employed for statistical analysis. We will estimate continuous values (including cognitive disorder changes, pain intensity, short-term memory, and quality of life) as mean difference or standardized mean difference and 95% confidence intervals (CIs), and dichotomous values (including incidence of adverse events) as risk ratio and 95% CIs. We will assess heterogeneity by checking the characteristics of eligible trials, disparities of subjects, types of interventions and comparators, and types of outcomes using *I*^2^ statistics. Its values will be interpreted as follows: 0% to 50% represents low heterogeneity; and 51% to 100% indicates considerable heterogeneity. A fixed-effect model will be used when there is low heterogeneity, while a random-effect model will be applied when there is obvious heterogeneity. If we identify low heterogeneity, we will carry out meta-analysis when sufficient studies involving the same outcome measurements are included, which focus on the similar study and patient characteristics, and interventions and controls. If we investigate considerable heterogeneity, we will carry out subgroup analysis to check the possible reasons for the obvious heterogeneity. Additionally, we will also merge the outcome data and report them as a narrative summary.

## 3 Discussion

A variety of published studies have reported that dexmedetomidine has been used for the management of CF in elderly patients after LCT. So far, no systematic review and meta-analysis has been conducted to evaluate the efficacy and safety of dexmedetomidine on CF in elderly patients after LCT. Therefore, it is very essential to investigate whether dexmedetomidine is effective and safety on CF in elderly patients after LCT. The findings of this study will provide helpful evidence for patients, clinician, as well as further studies.

## Author contributions

**Conceptualization:** Na Li, Lu Xiong, Xiao-Jian Chen, Ya-Zhen Meng, Shuang-Feng Li, Ya-Qin Xiong.

**Data curation:** Lu Xiong, Ye-hua Wu, Shuang-feng Li, Ya-qin Xiong.

**Formal analysis:** Na Li, Lu Xiong, Ye-Hua Wu, Ya-Zhen Meng, Ya-Qin Xiong.

**Funding acquisition:** Shuang-Feng Li.

**Investigation:** Shuang-Feng Li.

**Methodology:** Na Li, Lu Xiong, Ye-Hua Wu, Ya-Zhen Meng.

**Project administration:** Shuang-Feng Li, Ya-Qin Xiong.

**Resources:** Na Li, Lu Xiong, Ye-Hua Wu, Xiao-Jian Chen, Ya-Zhen Meng.

**Software:** Na Li, Lu Xiong, Ye-Hua Wu, Xiao-Jian Chen, Ya-Zhen Meng.

**Supervision:** Shuang-Feng Li, Ya-Qin Xiong.

**Validation:** Na Li, Lu Xiong, Xiao-Jian Chen, Ya-Zhen Meng, Shuang-Feng Li, Ya-Qin Xiong.

**Visualization:** Lu Xiong, Ye-Hua Wu, Xiao-Jian Chen, Ya-Zhen Meng, Shuang-Feng Li, Ya-Qin Xiong.

**Writing – original draft:** Na Li, Ye-Hua Wu, Ya-Zhen Meng, Shuang-Feng Li, Ya-Qin Xiong.

**Writing – review & editing:** Na Li, Lu Xiong, Xiao-Jian Chen, Shuang-Feng Li, Ya-Qin Xiong.
